# Reconstructing Foveola by Foveolar Internal Limiting Membrane Non-Peeling and Tissue Repositioning for Lamellar Hole-Related Epiretinal Proliferation

**DOI:** 10.1038/s41598-019-52447-4

**Published:** 2019-11-05

**Authors:** Tzyy-Chang Ho, Allen Yi-Lun Ho, Muh-Shy Chen

**Affiliations:** 1 Department of Ophthalmology, National Taiwan University Hospital, College of Medicine, National Taiwan University, No. 7, Chung-Shan S. Rd, Taipei City, 10002 Taiwan, ROC; 2 Department of Ophthalmology, Cardinal Tien Hospital, Fu Jen Catholic University, No. 362, Zhongzheng Rd., Xindian District, New Taipei City, 23148 Taiwan, ROC; 3 School of Medicine, College of Medicine, Fu-Jen Catholic University, New Taipei City, Taiwan, ROC; 4 School of Medicine, Chang Gung University, No. 259, Wenhua 1st Rd, Guishan District, Taoyuan City, 33302 Taiwan, ROC

**Keywords:** Retinal diseases, Vision disorders

## Abstract

Differences in the pathogenesis and clinical characteristics between lamellar macular hole (LMH) with and without LMH-associated epiretinal proliferation (LHEP) can have surgical implications. This study investigated the effects of treating LHEP by foveolar internal limiting membrane (ILM) non-peeling and epiretinal proliferative (EP) tissue repositioning on visual acuity and foveolar architecture. Consecutive patients with LHEP treated at our institution were enrolled. The eyes were divided into a conventional total ILM peeling group (group 1, n = 11) and a foveolar ILM non-peeling group (group 2, n = 22). In group 2, a doughnut-shaped ILM was peeled, leaving a 400-μm-diameter ILM without elevated margin over the foveola after EP tissue repositioning. The EP tissue was elevated, trimmed, and inverted into the LMH. Postoperatively, the LMH was sealed in all eyes in group 2, with significantly better best-corrected visual acuity (−0.26 vs −0.10 logMAR; p = 0.002). A smaller retinal defect (p = 0.003), a more restored ellipsoid zone (p = 0.002), and a more smooth foveal depression (p < 0.001) were achieved in group 2. Foveolar ILM non-peeling and EP tissue repositioning sealed the LMH, released the tangential traction, and achieved better visual acuity. The presumed foveolar architecture may be reconstructed surgically. LMH with LHEP could have a combined degenerative and tractional mechanism.

## Introduction

Pang *et al*.^1^ classified lamellar macular hole (LMH) into two types according to the findings on optical coherence tomography (OCT) as (1) associated with a high reflectivity epiretinal membrane (ERM) compared with the surface of the retina and (2) lamellar hole-associated epiretinal proliferation (LHEP) of homogenous medium reflectivity. Eyes with LHEP have been found to have poorer visual acuity, greater external LMH diameters, a thinner floor, and more anatomical disruption of the photoreceptor layer in the foveal region than those without LHEP^2–10^.

The aetiology of LMH is poorly understood. However, the conventional hypothesis is that it derives from contraction of an ERM that causes a tear in the inner retinal layers^11^. LHEP presents on spectral-domain OCT as an intermediate reflective material on the edge of a ruptured fovea^1^. During surgery, it may be noticed as a yellow elastic jelly that may toughen the ERM and internal limiting membrane (ILM) peeling^12^. In the absence of a clear aetiology for LHEP, the most likely theory is that it results from migration of retinal Müller glial cells^1^ to allow for “physiological” closure of the LMH. In one study, LHEP tissue stained positive for pan-keratin, which is a marker for retinal epithelial cells, leading the authors to hypothesise that LHEP originates in the retinal pigment epithelium^12^.

In spite of the anatomical differences between LMH with LHEP and LMH without LHEP, most studies have found no statistically significant difference in the prognosis either with observation or after surgical management^7,9,13^. One paper reported a worse visual outcome in patients with LMH and LHEP after surgery^8^, and another reported a positive correlation between the dimensions of the LMH and the area of the LHEP^14^. Postoperative retinal defect, poor recovery of the ellipsoid zone, and even formation of a macular hole have been reported^1,9,15^. It is possible that conventional total ILM peeling does not improve the surgical outcome because of poor reconstruction of the foveolar architecture.

The differences in pathogenesis and clinical characteristics between LMH with LHEP and LMH without LHEP are likely to have surgical implications. In this study, we reviewed the results of vitrectomy for ERM and ILM peeling for LMH with LHEP. We hypothesised that a rational approach of filling the retinal defect with EP tissue and preserving the foveolar ILM but peeling off the surrounding ILM using a foveolar ILM non-peeling technique may improve the surgical outcome.

## Results

There was no significant difference in age, sex, or duration of follow-up between the group that underwent conventional total ILM peeling (group 1) and the group that underwent foveolar ILM non-peeling (group 2; Table 1). No patient developed full-thickness macular hole during the postoperative follow-up period.

**Table 1:** Demographic and clinical characteristics of patients with macular lamellar hole and LHEP who underwent conventional ILM total peeling versus foveolar ILM non-peeling with proliferative tissue repositioning surgery.

Characteristic	Conventional ILM total peeling group (n = 11)	Foveolar ILM non-peeling with proliferative tissue repositioning group (n = 22)	*P-*value
Age (years)	67 ± 8.2	68 ± 9.0	0.285*
Female sex	11 (72.7%)	14 (70%)	0.492^†^
Preoperative BCVA (logMAR)	0.36 ± 0.25	0.38 ± 0.28	0.450*
Final BCVA (logMAR)	0.26 ± 0.28	0.12 ± 0.15	0.003*
Change in BCVA (logMAR)	−0.10 ± 0.35	−0.26 ± 0.22	0.002*
Intact preoperative ellipsoid zone (eyes)	8 (72.7%)	15 (68%)	0.465^†^
Intact postoperative ellipsoid zone (eyes)	8 (72.7%)	21 (95%)	0.002^†^
Preoperative retinal defect	11 (100%)	22 (100%)	0.462^†^
Postoperative retinal defect	6 (54.5%)	1 (4.5%)	0.003^†^
Preoperative smooth foveal depression	0 (0%)	0 (0%)	1^†^
Postoperative smooth foveal depression	1 (9%)	19 (86.4%)	<0.001^†^
Postoperative macular hole formation	0 (0%)	0 (0%)	1^†^
Follow-up duration (months)	28.2 ± 8.2	29.3 ± 10.1	0.823^†^

*Mann-Whitney *U* test; ^†^Fisher’s exact test; ^‡^Independent samples *t*-test. BCVA, best-corrected visual acuity; LHEP, lamellar hole-associated epiretinal proliferation; ILM, internal limiting membrane; logMAR, logarithm of the minimum angle of resolution

### Visual acuity

The logMAR (logarithm of the minimum angle of resolution) best-corrected visual acuity (BCVA) improved significantly from 0.36 ± 0.25 to 0.26 ± 0.28 (p = 0.021) in group 1 and from 0.38 ± 0.28 to 0.12 ± 0.15 (p < 0.001) in group 2. The amount of visual gain after surgery was significantly greater in group 2 than in group 1 (−0.10 ± 0.35 versus −0.26 ± 0.22, p = 0.002).

### Surgical outcomes

**Figure 1:**
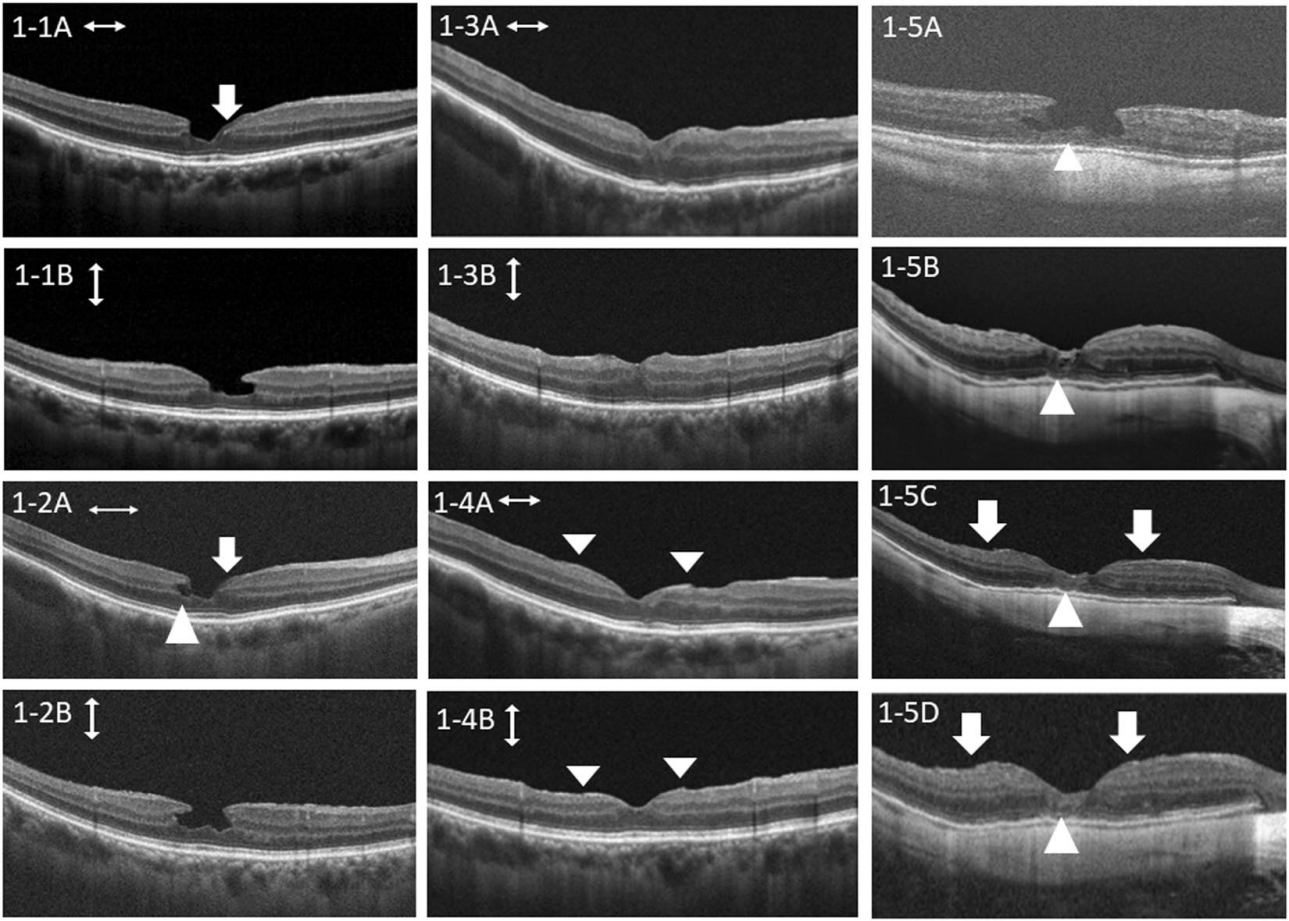
Case presentations. Figures 1-1–1-4 shows the case described in Fig. 4 and in the Supplementary Information). Figure 1-5A–1-5D shows a second case. The first was a 71-year-old man who had a lamellar macular hole (LMH) with lamellar hole-associated epiretinal proliferation (LHEP) in the right eye and a best-corrected visual acuity (BCVA) of 0.2 measured 2 years and 5 months preoperatively. The central foveal thickness (CFT) was 265 μm (1-1A, 1-1B). Epiretinal tissue is shown by the arrow. One month before surgery, a retinal defect was present (1-2A). The arrow shows the proliferative tissue. The BCVA had decreased to 0.1. The CFT had increased to 290 μm (1-2A, 1-2B). One month after foveolar internal limiting membrane (ILM) non-peeling and LHEP repositioning, the hole was well sealed with foveal depression (1-3A, 1-3B). The CFT was 317 μm. Three months postoperatively, the foveal depression was improved and the CFT had decreased to 258 μm (1-4A, 1-4B). The BCVA was improved to 0.8. The arrowheads mark the margin of the preserved ILM over the foveola (1-4A, 1-4B). The retinal defect was no longer present. A and B are horizontal and vertical optical coherence tomography scans, respectively. The second case (1-5A–1-5D) was a 56-year-old man who had LMH-associated epiretinal tissue with a disrupted ellipsoid zone (EZ) and external limiting membrane (arrowhead, 1-5A). He had a BCVA of 0.2 preoperatively. He underwent foveolar non-peeling internal limiting membrane (ILM) surgery with epiretinal tissue repositioning. Two months postoperatively, the LMH was well sealed with foveal depression, the EZ and external limiting membrane were growing toward the centre of the fovea, and there was a small retinal defect (arrowhead, 1-5B). The epiretinal proliferative tissue had been removed and the foveolar ILM was well preserved (the margins are marked by arrows, 1-5B). One further month later, the EZ and external limiting membrane has recovered almost completely and the retinal defect had disappeared (arrowhead, 1-5C). The BCVA improved to 0.4. Six months later, the BCVA improved to 1.0. The foveolar ILM was still well-preserved with a flat margin (arrows). The foveolar cone was restored gradually when compared with 1-5C with no retinal defect (1-5D). The EZ and external limiting membrane recovered (arrowhead).

**Figure 2:**
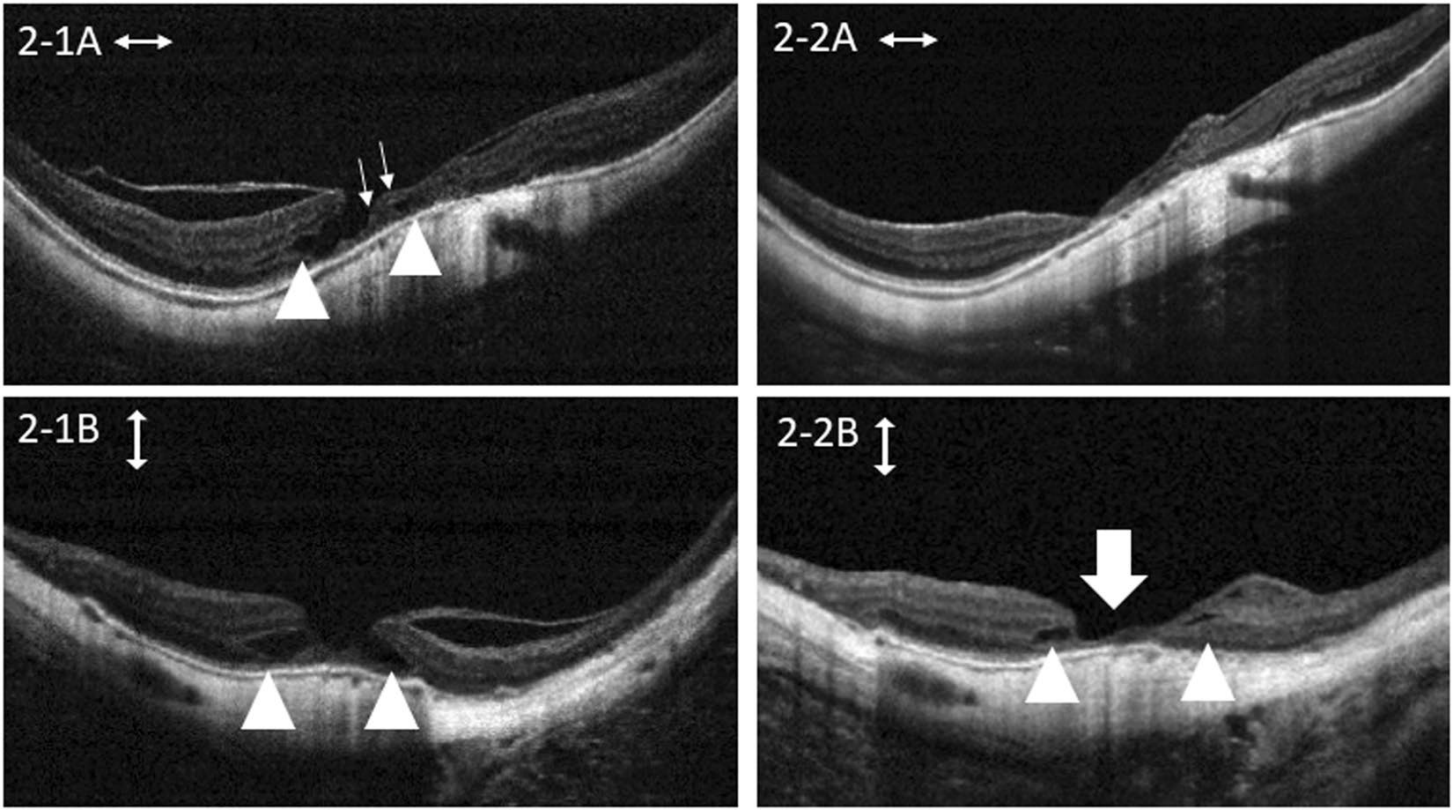
A 62-year-old woman with lamellar hole-associated epiretinal proliferative tissue and epiretinal membrane underwent pars plana vitrectomy with epiretinal tissue repositioning and total internal limiting membrane peeling. There was an outer retinal defect (arrowhead) and medium reflective epiretinal proliferative tissue (small arrows) before surgery (2-1A, 2-1B). Two years and 9 months postoperatively, there was a persistent outer retinal defect (arrowhead, 2-2A and 2-2B). The repositioned tissue was removed by total internal limiting membrane peeling, leaving a thin atrophic foveal structure (arrow). The medium reflective tissue in 2-1A was removed. A and B show horizontal and vertical optical coherence tomography scans at different time points.

**Figure 3:**
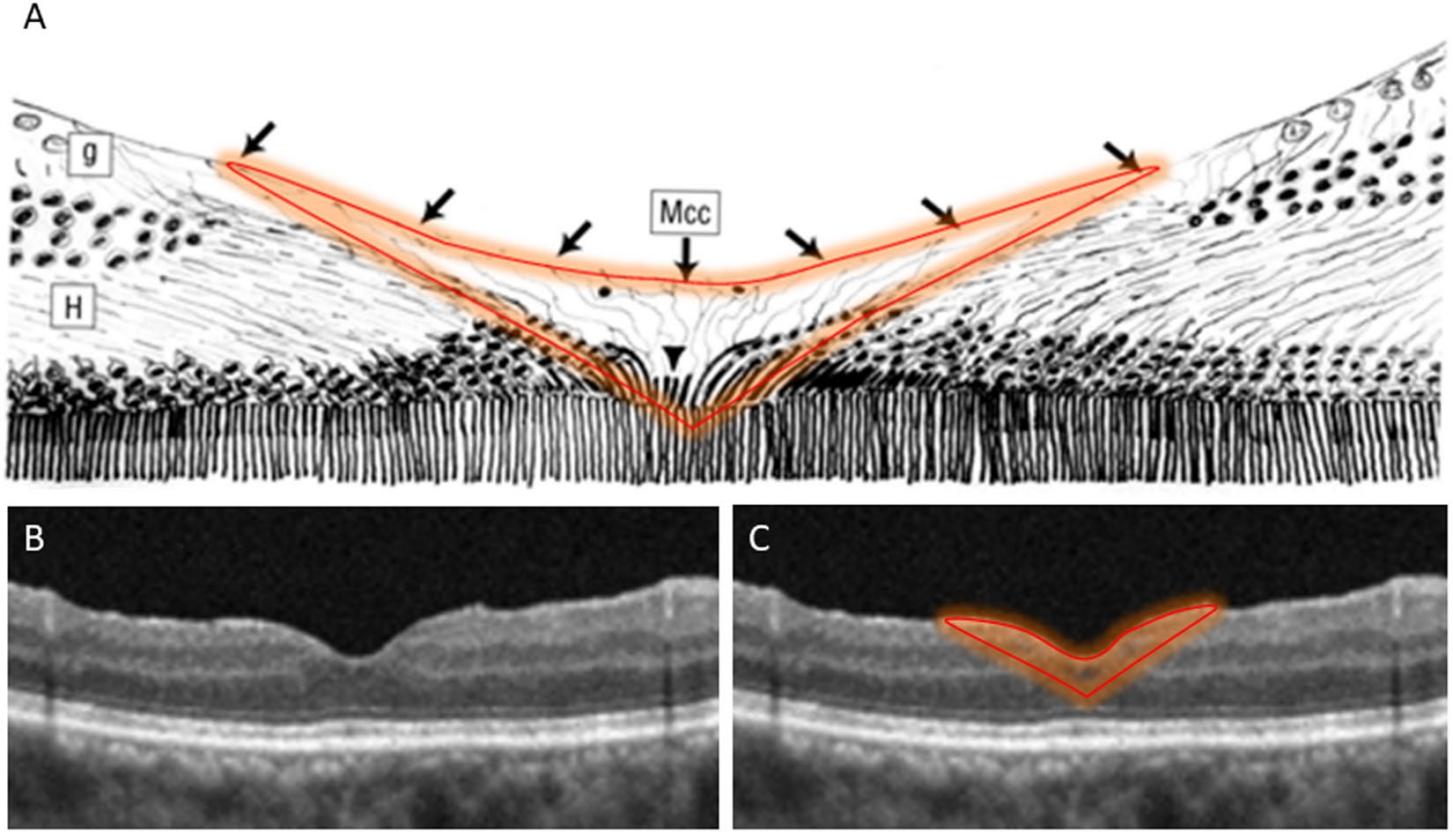
Schematic drawing of reconstruction of presumed Müller cell cones and foveolar architecture after foveolar internal limiting membrane non-peeling surgery in the case described in Fig. 1. (**A**) The orange outline denotes the Müller cell cones in the foveola originally described by Gass (1999) and Yamada (1969). (**B**) A postoperative OCT image of the case described in Fig. 1 without the Müller cell cones outlined. (**C**) A postoperative OCT image with the Müller cell cones outlined, highlighting reconstruction of Müller cell cones in the foveola. Mcc, Müller cell cones; g, ganglion cells; H, Henle’s fibres.

**Figure 4:**
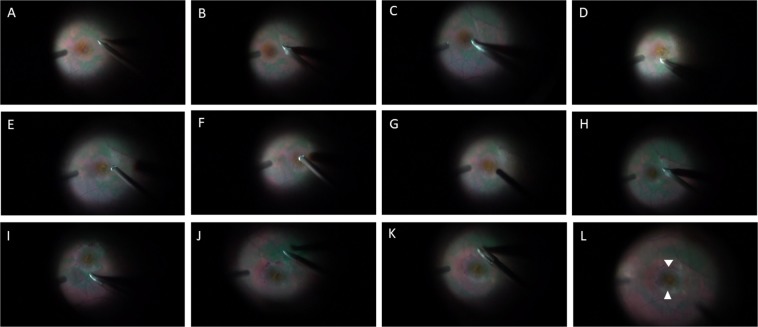
Serial photographs demonstrating the sequential surgical procedure of foveolar internal limiting membrane (ILM) non-peeling and epiretinal proliferative tissue repositioning surgery for epiretinal membrane and lamellar macular hole-associated epiretinal proliferative tissue. (**A**) Grasping the margin of the epiretinal membrane. (**B**) Starting to peel the epiretinal membrane over the lower margin (surgeon’s view). (**C**) Peeling the epiretinal membrane over the upper margin (surgeon’s view). (**D**) Completion of the peeling of the epiretinal membrane. (**E**) Initial trimming of the epiretinal membrane. (**F**) Tucking of the yellowish foveal tissue into the retinal cleavage. (**G**) Further trimming to an appropriate size. (**H**) Making a slit cut over the ILM. I. Peeling the upper part of the ILM in a circumferential manner (surgeon’s view). (**J**) Peeling to the lower part of the ILM (surgeon’s view). (**K**) Peeling the final part without elevation of the margin of the ILM. (**L**) A doughnut-shaped ILM is peeled off and the foveolar ILM is left over the foveola. The preserved foveolar margin of the ILM is shown by arrows.

The preoperative foveal OCT configurations showed no significant difference in the intact ellipsoid zone, retinal defect, or smooth foveal depression between the two groups (Table 1). Postoperative recovery of the ellipsoid zone was significantly better in group 2 than in group 1 (p = 0.002). In group 2, the persistent retinal defect was significantly reduced (p = 0.003) and more postoperative foveal depression was regained (p < 0.001). Figure 1-1 to 1-4 shows the case of a patient with LMH and LHEP in group 2 who underwent foveolar ILM non-peeling surgery with repositioning of LHEP tissue and regained smooth foveal depression without a retinal defect. Figure 1-5 shows the OCT images for a further patient 2 months and 6 months after foveolar ILM non-peeling with LHEP repositioning surgery and demonstrates gradual recovery of foveal depression, a preserved foveolar ILM, and recovery of the ellipsoid zone and the line of the external limiting membrane. The retinal defect disappeared gradually in this case. Figure 2 shows the case of a patient in group 1 who had poor recovery of the ellipsoid zone and a persistent retinal defect.

We observed 1 pseudophakic eye in group 1 and 2 pseudophakic eyes before the surgery. Phacoemulsification with intraocular lens implantation was performed in 2 eyes in group 1 and 4 eyes in group 2 because of the development of cataracts that hindered visual improvement after the vitrectomy surgery.

## Discussion

In this report we demonstrate that a combination of LHEP tissue repositioning and foveolar ILM non-peeling is an effective surgical treatment for LMH with LHEP. Our technique may provide a solution for the distinct entity of LMH with LHEP and may explain the variability in visual outcome previously reported after vitrectomy in patients who have LMH without LHEP. Previous reports have shown that conventional total ILM peeling produces worse final visual acuity and more postoperative disruption of the ellipsoid zone in eyes with LMH and LHEP than in eyes that have LMH without LHEP^8,13,16^.

There are two possible explanations for why this combination of surgical techniques is more effective than the LHEP repositioning technique alone for treating LMH with LHEP. The first explanation is that the foveolar ILM non-peeling technique ensures that the LHEP tissue remains securely in position after ILM peeling. If a conventional ILM peeling is performed, the repositioned tissue may be taken away completely or partially along with the ILM that has been peeled off. The second explanation is that this combination of surgical techniques might be more effective because the 360 degrees of tangential traction around the LMH is removed evenly. Given that LHEP is mainly composed of glial cells^6^, which are thought to play a major role in healing of the macula when repositioned into the retinal cleavage^17^, complete release of all the surrounding tangential traction would facilitate closure of the hole. The securely repositioned LHEP tissue and gas tamponade may act as a scaffold to promote healing of the glial cells. Our technique will ensure the preservation of the optimal amount of epiretinal proliferation left over during the operation. The traction from 360 degree around can be released completely and evenly so as to achieve a symmetric foveolar architecture. Both the management of epiretinal proliferative tissue and internal limiting membrane are essential in the restoration of foveolar architecture. In our study, smooth foveal depression was found in 86% of eyes treated by this technique.

In regard to the surgical technique, there has been a case report of an ILM inversion technique being used in combination with embedding of the LHEP^18^. When using that technique, an inverted ILM from upper to lower is made to cover the LMH after embedding the LHEP into the LMH. However, the tangential traction of the ILM surrounding the LMH is not released evenly by this technique. This is probably the reason why the foveal depression cannot be restored postoperatively. Furthermore, unlike with the technique devised by Shiraga *et al*.^17^, our technique involved the usage of an MVR blade to delineate the border of the non-peeling area when using our technique, which ensures preservation of the foveolar ILM. Shiraga’s technique described a method by peeling the ILM without delineating the border. In his surgical video he peeled the ILM to the hole margin supposing that the ILM was left behind. The technique was likely to peel off some or all of the internal limiting membrane as well as some of the preserved proliferative tissue.

Recent advances in OCT have revealed that LMH can be classified into two types based on the presence or absence of pathological traction^19,20^. LMHs with pathological retinal traction are classified as tractional LMHs and those without it are classified as degenerative LMHs. LHEP has been thought to be found only in degenerative LMH, with one study reporting that LHEP was present in 80% of eyes with this type of LMH^20^. However, in our study, we found that if the tangential traction of all the ILM surrounding the LMH is released, the foveal depression can be restored evenly with a decrease in central foveal thickness (Fig. 1). Moreover, our present findings suggest that there is a tractional component in the pathogenesis of LMH. If only degenerative component is involved in the pathogenesis the release of the internal limiting membrane traction would not be necessary. The OCT images show incompliance of the inner retinal surface before surgery and released incompliance after surgery (e.g. comparing 1-1 B and 1-4B).

Unlike in some previous reports^1,13^, we did not find a persistent postoperative retinal defect. There are two possible reasons for this: first, our technique released the tangential ILM traction evenly, allowing for even recovery of the preoperative retinal defect, and second, the repositioned LHEP tissue replaced the missing tissue in the retinal cleavage and blocked repair of glial cells after complete release of the tangential ILM traction. This effect had postoperative benefits following reconstruction of the foveolar architecture in our series.

The fovea itself is a 1500 μm depression in the centre of the macula. The central 350 μm of the fovea (known as the foveola) is located in a retinal capillary-free zone that has a diameter of about 500 μm^21–23^. Only photoreceptor (cone) cells and Müller cell processes are present in the foveolar area. Preservation of the foveolar ILM maintains the expanded vitreal processes of the Müller cell. The integrity of the fine processes of the Müller cells that envelop the neurons can be preserved to the greatest extent possible using our technique. Figure 3 shows a schematic drawing of the presumed Müller cell cones and foveolar architecture after reconstruction surgery as in the case shown in Fig. 1. The orange outline in Fig. 3A shows the Müller cell cones in the foveola first identified by Gass^22^. Figure 3B shows an OCT image acquired postoperatively for the case shown in Fig. 1 without the Müller cell cones outlined, and Fig. 3C shows a postoperative OCT image for the same patient with the Müller cell cones outlined, highlighting the reconstruction of Müller cell cones in the foveola. The schematic drawing shown in Fig. 3C underscores the critical importance of reconstruction of the presumed foveolar architecture in recovery of foveal depression and the layered structures and microstructures of the retina in the surgical treatment of this disease entity.

Morescalchi *et al*.^24^ described a technique of peeling of the ILM with foveal sparing for treatment of degenerative lamellar macular hole. The technique adopted by Morescalchi F is different from our foveolar nonpeeling technique. Their technique is similar to Shimada’s^25^ and involves an ILM peeling starting from further away from fovea and peeled to the centre and trimming the floating flap of ILM to the size of one to two disc size. Their technique leaves the margin elevated and is much larger than ours (our size is smaller than the fovea avascular zone and is approximately 400 μ in diameter). Our technique leaves the foveolar ILM as a foveolar size and a flat margin. Our foveolar ILM nonpeeling is critical in reconstructing the foveolar architecture as a U-shaped foveal depression and layered retinal structures. In Morescalchi’s article the cases they presented were shown to have extra and unsmooth proliferative tissue, retinal defect by OCT and micro-scotomas by retinal sensitivity testing post-operatively. The follow-up duration in their study was 6 months. We found that the foveolar structures restored and maintained after an average of follow-up period of 28 months.

There are several limitations to this report, stemming mainly from its retrospective observational design. Other limitations included the relatively smaller number of cases in group 1 and the intact ellipsoid zone, retinal defect, and smooth foveal depression are evaluated subjectively. Furthermore, we have described a technique that probably entails a learning curve. However, we have provided serial surgical photographs (Fig. 4) and a surgical recording (Supplementary Information, Video S1) for reference.

In conclusion, our results suggest that combining the LHEP repositioning technique with foveolar ILM non-peeling might be an effective treatment for LMH with LHEP. The findings of this research also suggest that ILM traction exists in this disease entity, despite it having been thought to be degenerative in nature.

## Patients and Methods

In this observational case series, we retrospectively reviewed the medical records of 31 consecutive patients who underwent vitrectomy for LMH with LHEP at our institution from January 2013 to December 2016. The study was approved by the National Taiwan University Hospital Research Committee and adhered to the tenets of the Declaration of Helsinki. All patients who participated in the study gave written informed consent after receiving an explanation of the nature of the study and its possible consequences.

LHEP was identified on spectral-domain OCT images (Cirrus HD-OCT, Carl Zeiss Meditec, Inc., Dublin, CA, or RTVue Model-RT 100; version 3.5, Optovue, Inc., Fremont, CA) based on the characteristics described by Pang *et al*.1. OCT studies were conducted every 2-6 months. Patients with other macular diseases, retinal vascular diseases, hereditary macular diseases or previous vitreoretinal surgeries were excluded. Patients who had LMH with LHEP underwent surgery if best-corrected visual acuity (BCVA) was worse than 20/40. The eyes were divided into 2 groups: Group 1 (11 eyes) received vitrectomy, EP tissue repositioning, total ILM peeling, and air-fluid exchange, and Group 2 (22 eyes) received vitrectomy, a doughnut-shaped ILM was peeled, leaving a 400-μm-diameter ILM over the foveola after EP tissue repositioning, and air-fluid exchange.

Basic demographic data were collected retrospectively by chart review for each patient. The data collected included age, sex, surgical history, operative methods used, and duration of follow-up. BCVA measurements were made using a Snellen acuity chart and converted to the logMAR scale for all data analyses.

### Surgical technique

A standard three-port pars plana vitrectomy was performed in all cases by the same surgeon (TCH) using a 23-gauge or 25-gauge vitrectomy system. After core vitrectomy, the posterior hyaloid was completely removed with or without the assistance of triamcinolone acetonide. Diluted indocyanine green dye (initially 25 mg in 20 mL of 5% glucose and diluted 10 further times with 5% glucose) was then used to stain the ILM for 30 seconds. We then applied a surgical technique whereby the ERM is removed by picking up the margin between the non-stained ERM area and the stained ILM area using a bent MVR blade. After peeling to the margin of the LMH, the yellowish LHEP material is left unpeeled. The LHEP material is then trimmed with a 23-gauge or 25-gauge vitrectomy cutter to an appropriate size to fill the retinal defect. A foveolar ILM non-peeling technique is then performed with the aid of an MVR blade, microscissors and forceps to leave a 400-μm-diameter ILM over the foveola (see Fig. 4 and Video S1 in the Supplementary Information) with a preserved ILM margin that is not elevated. The authors have used a similar surgical technique to treat early stage 2 macular hole and myopic traction maculopathy^26,27^. At the end of surgery, air-fluid exchange and 15% perfluoropropane (C3F8) were used as gas tamponade. All subjects were advised to maintain a prone position for 7 days. In the total peeling group the LHEP material is elevated and saved as much as possible. Conventional total ILM peeling is then performed without preserving the foveolar ILM.

### Statistical analysis

The Mann-Whitney U test was used to compare the preoperative BCVA, postoperative BCVA, and perioperative BCVA gain. Wilcoxon’s signed-rank test was used to compare the preoperative and postoperative BCVA in each study group. The statistical analyses were performed using SPSS version 22.0 software (IBM Corp., Armonk, NY). Statistical significance was defined as p < 0.05.

## Supplementary information

Foveolar ILM nonpeeling surgery of LHEP

## Data Availability

The datasets generated during and/or analysed during the current study are available from the corresponding author on reasonable request.
